# *Cylindrocarpon lichenicola* keratomycosis in Nigeria: the challenge of limited access to effective antimicrobials

**DOI:** 10.4102/ajlm.v6i1.612

**Published:** 2017-07-11

**Authors:** Emmanuel O. Irek, Temitope O. Obadare, Patrick A. Udonwa, Olajumoke Laoye, Oyekola V. Abiri, Adenike O. Adeoye, Aaron O. Aboderin

**Affiliations:** 1 Department of Medical Microbiology and Parasitology, Obafemi Awolowo University Teaching Hospitals Complex, Ile-Ife, Osun state, Nigeria; 2 Department of Ophthalmology, Obafemi Awolowo University Teaching Hospitals Complex, Ile-Ife, Osun state, Nigeria

## Abstract

**Introduction:**

We report a rare cause of keratitis, due to *Cylindrocarpon lichenicola*, in a farmer with keratomycosis. Despite the acknowledged virulence of this fungus, a suitable antifungal for its management was not accessible.

**Case presentation:**

A 67-year-old farmer presented with a two-week history of pain, mucopurulent discharge, redness and a corneal ulcer with a visual acuity of hand movement in the right eye. With a working diagnosis of infective keratitis, corneal scrapings were taken under a slit lamp biomicroscope for microbiological testing. Direct lactophenol cotton blue mounts revealed septate fungal hyphae, while fungal culture on Sabouraud dextrose agar at room temperature grew woolly mould phenotypically consistent with *C. lichenicola*.

**Management and outcome:**

The patient was started on hourly topical natamycin (5%), ciprofloxacin (0.3%), two-hourly instillation of tobramycin (0.3%) and atropine (1%) twice daily for three months following the isolation of the fungus. The eye healed with a corneal scar and no improvements in visual acuity.

**Discussion:**

This infection was difficult to manage due to the inaccessibility of a suitable antifungal, namely, voriconazole in our setting. Hence, there is a need for prompt identification and early institution of suitable antifungals in any patient with suspected keratomycosis.

## Introduction

Filamentous fungi are an emerging cause of keratitis worldwide,^1^ including occurrences of some rarer fungi. Only a few cases of keratitis due to *Cylindrocarpon lichenicola* have been reported in published literature in the world, none of these in sub-Saharan Africa. The occurrence of keratomycosis is, however, partly associated with farming,^2^ which is a predominant occupation in the tropics. Hence, keratomycosis from plant or soil particles laden with fungal materials is not uncommon in these areas.^3^ *C. lichenicola* is a hyaline filamentous fungus which is also called *Fusarium lichenicola* and has been reclassified as a member of the *Fusarium solani* species complex.^1^ However, it rarely causes keratitis,^4^ although it has been implicated in cutaneous mycosis in immunocompetent patients.^5,6^ It has devastating effects on the eye following infection, despite use of suitable antifungals.^3^ In this article, we describe fungal keratitis caused by *C. lichenicola* in a man living in a semi-urban region in Nigeria.

## Ethical considerations

### Informed consent

Written informed consent was obtained from the patient after adequate explanation was provided and confidentiality was assured.

### Data protection

The patient’s identification data were stored on a password protected computer accessible only to the authors and the managing team.

## Case presentation

A 67-year-old male farmer presented to the Ophthalmology Unit of Obafemi Awolowo University Teaching Hospitals Complex in Ile-Ife, Nigeria, with a two-week history of pain, mucopurulent discharge and redness in the right eye. There was no history of foreign body entry into the right eye, nor was there ocular trauma or instillation of traditional eye medication. The patient had earlier used chloramphenicol eye drops which he obtained over the counter. His fasting blood sugar, complete blood count and electrolyte urea and creatinine were essentially normal for his age. The patient’s HIV status was negative on serology testing.

**FIGURE 1:**
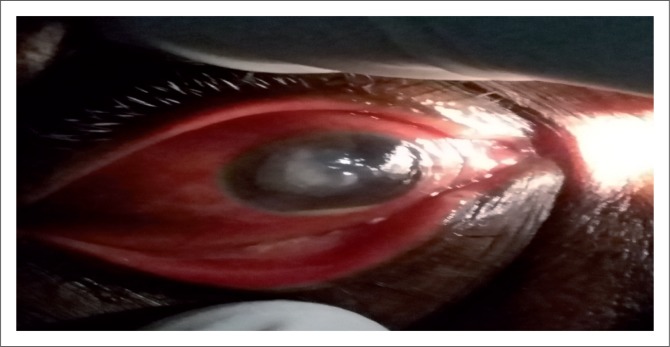
Right eye examination showing hypopyon, corneal ulcer measuring 5.5 x 4 mm with irregular margins.

**FIGURE 2:**
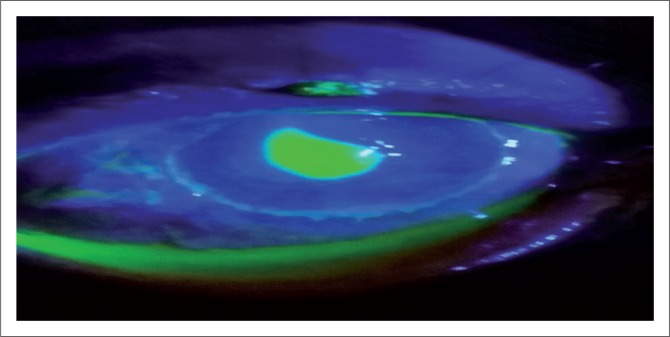
Right eye examination under slit lamp with fluorescein dye highlighting the corneal ulcer.

**FIGURE 3:**
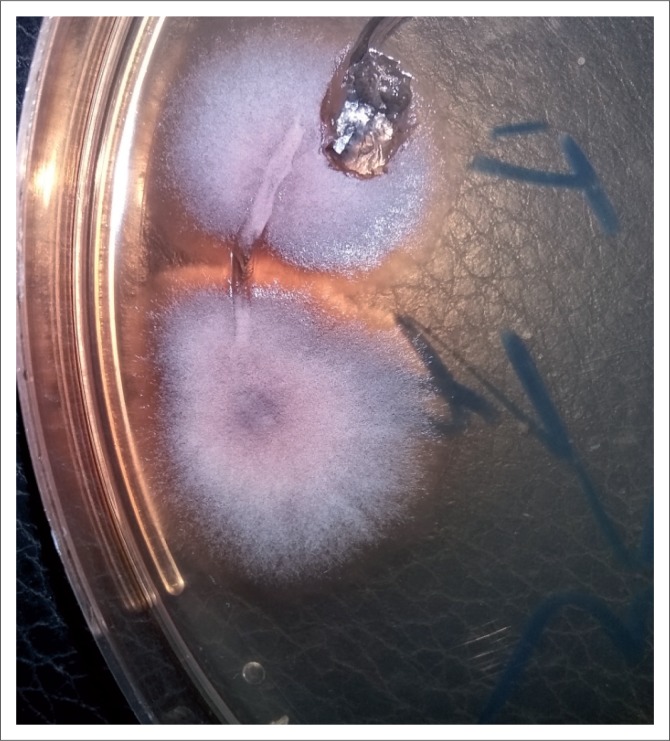
Woolly mould with reddish brown pigmentation after culture of corneal scraping on Sabouraud dextrose agar at room temperature for 48 hours.

**FIGURE 4:**
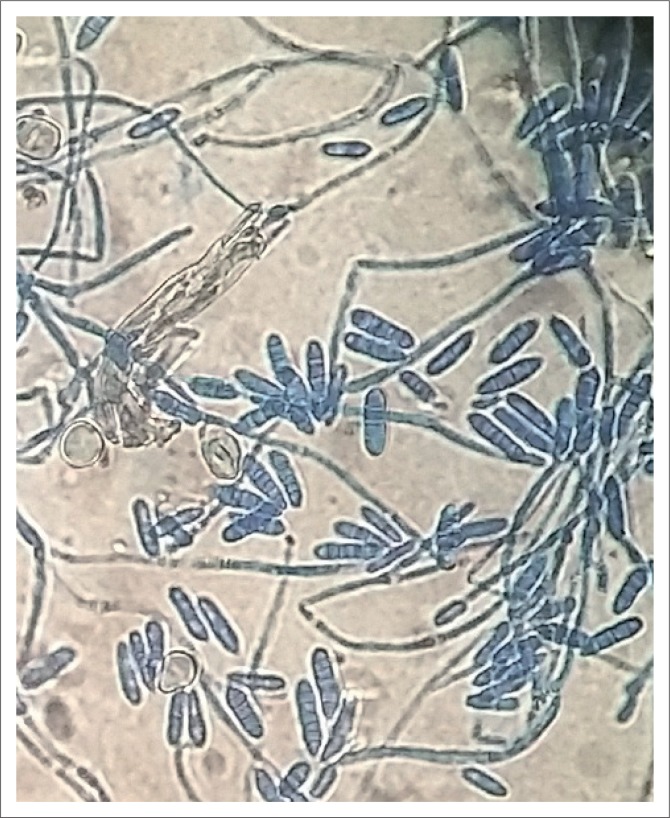
*Cylindrocarpon lichenicola* chlamydospores, conidiophores and conidia stained with lactophenol phenol cotton blue (x400 magnification).

Further, ocular examination revealed a visual acuity of hand movement in the right eye, unaided and aided. Slit lamp biomicroscopic examination of the right eye showed a diffuse conjunctival hyperemia and a 5.5 x 4 mm corneal ulcer with raised and irregular margins (Figure 1) which stained with fluorescein (Figure 2). Whitish stromal infiltrates were present in the ulcer bed and around the ulcer margins with associated stromal oedema and folds in the Descemet’s membrane. Anterior chamber examination revealed hypopyon of about one-eighth of the anterior chamber height. Pupil was round but sluggishly reactive to light and there was early lens opacity and no glow on fundoscopy in the right eye. Ocular findings in the left eye were essentially normal. A presumptive diagnosis of infective keratitis was made. During the slit lamp biomicroscopy, corneal scrapings were taken from the margins and the base of the ulcer and were sent to the microbiology and parasitology laboratory for bacterial and fungal tests. Direct lactophenol cotton blue mounts revealed septate fungal hyphae, while direct Gram stain showed cellular debris but no microorganisms. Culture on chocolate agar yielded scanty growth of cottony white colonies after 48 hours of incubation at 37°C. However, culture on Sabouraud dextrose agar at room temperature supported growth of woolly mould with reddish brown pigmentation on the agar after 48 hours (Figure 3). Lactophenol cotton blue staining of the mould under light microscope (x400 magnification) revealed conidiophores consisting of phialides, arranged in brush-like structures. Moreover, the phialides were cylindrical with small collarettes producing hyaline, smooth-walled conidia, which were arranged in masses. The macroconidia were septate, cylindrical with rounded apex and flat base^7^ (Figure 4). No bacteria were seen. The morphology of the mould identified was consistent with *C. lichenicola*.^7^

## Management and outcome

This patient was started on hourly topical natamycin (5%), ciprofloxacin (0.3%), two-hourly instillation of tobramycin (0.3%) and atropine (1%) twice daily. He was also placed on oral fluconazole (200 mg) daily, 250 mg of acetazolamide daily and oral analgesics, all for three months following the isolation of the fungus. Despite the duration of use of the medication, the patient’s vision did not improve, as the visual acuity remained hand movement in the right eye (unaided and aided) and healed with a corneal scar.

## Discussion

Keratomycosis caused by *C. lichenicola* is rare in humans and challenging to manage. Although voriconazole has been used with success in some reports in managing *C. lichenicola*,^8^ its accessibility in our setting is limited. Generally, there is lack of access to some specific antimicrobial agents needed for possible successful treatment of infections (such as this) in low- and middle-income countries such as Nigeria.^9^ Although the circumstance following the occurrence of the fungus in this patient could not be fully ascertained, farming has been associated with keratomycosis.^2^

Prompt diagnosis with early institution of medication for keratomycosis caused by *C. lichenicola* is therefore needed to avert debilitating effects on the eye, such as were seen in this patient, or visual loss as reported in literature.^3^ Moreover, appropriate access to effective antimicrobials, in this circumstance voriconazole, in low- and middle-income countries is essential while restricting inappropriate use (which drives antimicrobial resistance).

Although keratomycosis caused by *C. lichenicola* has been reported in different parts of the world,^4,8,10^ this appears to be the first report of such in published literature from sub-Saharan Africa.
